# Development of a Rapid Reverse Blot Hybridization Assay for Detection of Clinically Relevant Antibiotic Resistance Genes in Blood Cultures Testing Positive for Gram-Negative Bacteria

**DOI:** 10.3389/fmicb.2017.00185

**Published:** 2017-02-09

**Authors:** Hye-young Wang, Gilsung Yoo, Juwon Kim, Young Uh, Wonkeun Song, Jong Bae Kim, Hyeyoung Lee

**Affiliations:** ^1^Optipharm M&D, Inc., Wonju Eco Environmental Technology CenterWonju, Gangwon, South Korea; ^2^Department of Laboratory Medicine, Yonsei University Wonju College of MedicineWonju, South Korea; ^3^Department of Laboratory Medicine, Hallym University College of MedicineSeoul, South Korea; ^4^Department of Biomedical Laboratory Science, College of Health Sciences, Yonsei UniversityWonju, South Korea

**Keywords:** extended-spectrum β-lactamase, AmpC β-lactamase, carbapenemase, REBA, qPCR, blood culture

## Abstract

Rapid and accurate identification of the causative pathogens of bloodstream infections is crucial for the prompt initiation of appropriate antimicrobial therapy to decrease the related morbidity and mortality rates. The aim of this study was to evaluate the performance of a newly developed PCR-reverse blot hybridization assay (REBA) for the rapid detection of Gram-negative bacteria (GNB) and their extended-spectrum β-lactamase (ESBL), AmpC β-lactamase, and carbapenemase resistance genes directly from the blood culture bottles. The REBA-EAC (ESBL, AmpC β-lactamase, carbapenemase) assay was performed on 327 isolates that were confirmed to have an ESBL producer phenotype, 200 positive blood culture (PBCs) specimens, and 200 negative blood culture specimens. The concordance rate between the results of REBA-EAC assay and ESBL phenotypic test was 94.2%. The sensitivity, specificity, positive predictive value, and negative predictive value of the REBA-EAC assay for GNB identification in blood culture specimens were 100% (95% CI 0.938–1.000, *P* < 0.001), 100% (95% CI 0.986–1.000, *P* < 0.001), 100% (95% CI 0.938–1.000, *P* < 0.001), and 100% (95% CI 0.986–1.000, *P* < 0.001), respectively. All 17 EAC-producing GNB isolates from the 73 PBCs were detected by the REBA-EAC assay. The REBA-EAC assay allowed easy differentiation between EAC and non-EAC genes in all isolates. Moreover, the REBA-EAC assay was a rapid and reliable method for identifying GNB and their β-lactamase resistance genes in PBCs. Thus, this assay may provide essential information for accelerating therapeutic decisions to achieve earlier appropriate antibiotic treatment during the acute phase of bloodstream infection.

## Introduction

Antibiotic resistance among pathogenic organisms has become a major healthcare problem worldwide, with serious consequences for the treatment of infectious diseases (Gottlieb and Nimmo, 2011). Early targeted antibiotic treatment is an important prognostic factor, especially for seriously ill patients (Cosgrove, 2006). Among the GNB, the Enterobacteriaceae family includes the most widespread pathogens causing infectious diseases; its members are among the most common causes of nosocomial infection. Beta-lactamase production is the major resistance mechanism of the Enterobacteriaceae. Specifically, extended-spectrum β-lactamases (ESBLs), plasmid-mediated cephalosporinases (pAmpCs), and carbapenemases pose major therapeutic challenges in the treatment of hospitalized and community-based patients. Rapid and accurate identification of β-lactamase-producing Enterobacteriaceae is crucial for reducing clinical failure due to the use of inappropriate antimicrobial therapy and for preventing nosocomial outbreaks.

The CLSI has released recommendations for the detection of ESBLs in clinical isolates of *Klebsiella pneumoniae, Klebsiella oxytoca, Escherichia coli*, and *Proteus mirabilis* using clavulanic acid (CLSI, 2015). However, no guidelines have yet been issued for the detection of AmpCs. Moreover, the occurrence of bacteria that co-produce ESBLs and AmpCs has become a serious issue in the context of Enterobacteriaceae β-lactam resistance. Furthermore, overproduction of AmpCs in association with a porin defect can lead to carbapenem resistance (Jacoby, 2009). Although the modified Hodge test for carbapenemases has been found to be a useful tool for the detection of carbapenemase-producing Enterobacteriaceae, this test lacks sensitivity for NDM carbapenemases. Moreover, false positive results can occur in isolates that produce ESBL or AmpC enzymes coupled with porin loss.

Currently, molecular diagnostic methods such as real-time PCR (qPCR) (Beuving et al., 2011), mass spectrometry (Chong et al., 2015), microarray (Card et al., 2013), and loop-mediated isothermal amplification-based systems (García-Fernández et al., 2015) are available for bacterial identification and discrimination of antimicrobial susceptibility. However, DNA-based ESBL identification is technically challenging because new ESBLs are derived from pre-existing class A β-lactamases by several single amino acid substitutions (Rubtsova et al., 2010).

In this study, we evaluated a PCR-based reverse blot hybridization assay (REBA) that was recently developed for the rapid detection of GNB and their ESBL, AmpC, and carbapenemase (EAC) resistance genes. Specifically, the REBA-EAC assay was designed to detect ESBLs (CTX-M-, TEM-, and SHV-type), AmpCs (ACT, CMY-2-like, DHA, ACC-1, CMY-1-like/MOX, and FOX), and carbapenemases (IMP, VIM, NDM, KPC, OXA-48-like, and SPM).

## Materials and methods

### Archived isolates

To determine the specificity of the REBA-EAC assay, we tested archived frozen stocks of 29 EAC-producing Enterobacteriaceae strains (Song et al., 2015) and 128 non-EAC-producing clinical isolates belonging to 35 different genera [*Citrobacter* (1), *Enterobacter* (2), *Escherichia* (9), *Klebsiella* (3), *Leclercia* (1), *Proteus* (3), *Salmonella* (5), *Serratia* (1), *Shigella* (4), *Bordetella* (1), *Haemophilus* (1), *Acinetobacter* (1), *Pseudomonas* (2), *Enterococcus* (17), *Micrococcus* (1), *Staphylococcus* (12), *Streptococcus* (5), *Corynebacterium* (1), *Mycobacterium* (23), *Absidia* (1), *Aspergillus* (7), *Aureobasidium* (1), *Beauveria* (1), *Bipolaris* (1), *Candida* (8), *Cryptococcus* (1), *Curvularia* (1), *Epidermophyton* (1), *Fusarium* (1), *Kodamaea* (1), *Malassezia* (1), *Microsporum* (3), *Penicillium* (2), *Saccharomyces* (1), and *Trichophyton* (4)] from various specimen types. The 29 EAC-producing Enterobacteriaceae isolates included 11 ESBL-producing, 9 AmpC-producing, and 9 carbapenemase-producing strains (Table 1).

**Table 1:** **Characteristics of the 29 EAC-producing Enterobacteriaceae isolates with ESBL, AmpC β-lactamase, and carbapenemase resistance genes**.

**Organism type (no. of isolates)**	**Stock no**.	**Resistance gene**
**ESBL STRAINS (11)**		
*Escherichia coli*	DML1101	CTX-M-1
*E. coli*	DML1786	CTX-M-1
*E. coli*	DML1100	CTX-M-9
*E. coli*	DML1780	SHV-2a
*E. coli*	DML1783	SHV-2a
*E. coli*	DML1784	SHV-151
*E. coli*	DML1785	SHV-28
*Klebsiella pneumoniae*	DML1098	CTX-M-1
*K. pneumoniae*	DML1095	SHV-12
*K. pneumoniae*	DML1098	SHV-28
*Shigella* spp.	DML1096	TEM-52
**AmpC** β**-LACTAMASES (9)**		
*E. coli*	DLMH 71	CMY-2-like
*E. coli*	DML1784	CMY-11
*E. coli*	DLMH 173	CMY-1-like/MOX
*E. coli*	DLM 256	ACC-1
*K. pneumoniae*	DLMH 70	DHA-1
*K. pneumoniae*	DLMH 585	DHA-1
*K. pneumoniae*	DLMH 492	CMY-1-like/MOX
*Enterobacter cloacae*	DML1099	ACT
*E. cloacae*	DML1781	ACT
**CARBAPENEMASES (9)**		
*K. pneumoniae*	DLMH 411	OXA-232
*K. pneumoniae*	DLMH 107	OXA-48-like
*K. pneumoniae*	DLMH 108	OXA-181
*K. pneumoniae*	DLMH 492	NDM
*E. coli*	DLMH 410	OXA-232
*E. coli*	DLMH 744	KPC
*E. cloacae*	DML 1781	VIM
*E. cloacae*	DLMH 568	VIM
*Pseudomonas aeruginosa*	DLMH 155	IMP

*DML, Diagnostic Microbiology Laboratory of Yonsei University (Wonju, Republic of Korea); DLMH, Department of Laboratory Medicine of Hallym University College of Medicine (Seoul, Republic of Korea)*.

### Clinical isolates

To assess the diagnostic performance of the REBA-EAC assay, 327 ESBL phenotype clinical isolates obtained from 2010 to 2014 at Wonju Severance Christian Hospital (WSCH) were used. Isolates were identified by conventional identification method including phenotypic characteristics such as colony morphology, Gram staining result, and commercial identification kit results. MicroScan (Beckman Coulter, Brea, CA, USA) overnight Pos BP Combo 28, MICroSTREP Plus, overnight Neg Combo 53 and Neg Combo 54 panels were used for the identification of GPB, streptococci and GNB. For the identification of *Candida* spp., VITEK 2 (bioMérieux, Durham, NC, USA) yeast identification card was used. The ESBL phenotype of each isolate was confirmed by the microbroth dilution method, which is the test recommended by the CLSI. AmpC β-lactamase and carbapenemase production was confirmed using qPCR and by sequencing analyses with EAC primers (Table 2). This study was approved by the Institutional Ethics Committee at Yonsei University Wonju College of Medicine (approval no. CR312055-002) and samples were de-identified.

**Table 2:** **Primers and probes for REBA-EAC and qPCR**.

**Target and primer/probe name**	**Nucleotide sequence (5′-3′)**	**Product size**
**ESBLs**		
CTX-M-1-210-F	GATGTGCAGCACCAGTAAAGTGATG	203 bp
CTX-M-1-413-R	ATCGCCACGTTATCGCTGTACTG	
CTX-M-1/2-258-P	AAGTGAAAGCGAACCGAATCTGTTA	
CTX-M-9-210-F	TGTGCAGTACCAGTAAAGTTATGGCG	170 bp
CTX-M-9-380-R	GCGCTAAGCTCAGCCAGTGACA	
CTX-M-9-261-P	GAAACGCAAAAGCAGCTGCTT	
TEM-520-F	TGCTCACCCAGAAACGCTGGT	705 bp
TEM-1225-R	ACGATACGGGAGGGCTTACCAT	
Gln39Lys-P	AAAGATGCTGAAGATAAGTTGGGTG	
Glu104Lys-P	CCAACTATCAAGGCGGGTTACA	
Gly164His-P	TTCCCAATGATCAAGGCGAGTTA	
Gly238Ser-P	GAGATCCATGCTCACCGGCTC	
Glu240Lys-P	GAGATCCATGCTCACCGGCTC	
TEM-127-F^a^	GGTTACATCGAGCTGGATCTCAACA	360bp
TEM-488-R^a^	CAACGATCAAGGCGGGTTACAT	
SHV-243-F	TATTATCTCCCTGTTAGCCACCCT	717 bp
SHV-960-R	CCAAGCAGGGCGACAATCC	
Leu35Gln-P	GGCTTTCGCTTTGTTTAATTTGCTC	
Asp179Ala-P	GCCCGCACCACCACTA	
SHV-147-F^b^	GCAAATTAAACAAAGCGAAAGCCA	133bp
SHV-280-R^b^	GCACTACTTTAAAGGTGCTCATCATGG	
**AmpC** β**-LACTAMASES**		
ACT-MIR-416-F	TACAGGTRCCGGATGAGGTCACG	205 bp
ACT-MIR-620-R	CTATGGTCCAGCTTGAGCGGCTTAA	
ACT-MIR-464-P	ATCAAAACTGGCAGCCGCAG	
CMY-2-like-261-F	AAGACGTTTAACGGCGTGTTGG	175 bp
CMY-2-like-436-R	TAACGTCATCGGGGATCTGCA	
CMY2-like-328-P	ACGAAATACTGGCCAGAACTGACA	
DHA-261-F	TTTCACAGGTGTGCTGGGTGC	200 bp
DHA-460-R	AGAAATTCAGCAGATCCGCACGG	
DHA-299-P	AAGAGATGGCGCTGAATGATCC	
ACC-1-410-F	ACGT AGCGTAACAAGTCGCTGGAG	210 bp
ACC-1-620-R	TGGGTGTGAGCTATGAAGATGCGATT	
ACC-1-439-P	GCTTCGTTACCCAAAATCTCCATATTC	
CMY-1-like/MOX-128-F	CTGCTCAAGGAGCACMGGATC	243 bp
CMY-1-like/MOX-371-R	CGGATCCCTTGAGCCAGGG	
CMY-1-like/MOX-172-P	AAAGGATGGCAAGGCCCACTA	
FOX-182-F	ATTTCAACTATGGGGTTGCCAACC	208 bp
FOX-390-R	AAGCTCGGCCATRGTCACACCATC	
FOX-239-P	TGTTCGAGATTGGCTCGGTCAG	
**CARBAPENEMASES**		
IMP-263-F	TAAAAGGCAGTATYTCCTCWCATTT	232 bp
IMP-495-R	CCAAACCACTACGTTATCTKGAG	
IMP-307-P	GGAATAGAGTGGCTTAATTCTCRATCTAT	
VIM-136-F	CTTTACCAGATTGCCGATGGTGTT	214 bp
VIM-350-R	TCATGAAAGTGCGTGGAGACTGCA	
VIM-198-P	CTACCCGTCCAATGGTCTCATTGT	
KPC-269-F	TGGACACACCCATCCGTTACG	211 bp
KPC-480-R	ACGGAACGTGGTATCGCCGATAG	
KPC-329-P	AATATCTGACAACAGGCATGACGGT	
NDM-327-F	AGATCCTCAACTGGATCAAGCAGG	223 bp
NDM-550-R	TGCTGGTTCGACCCAGCCATTGGC	
NDM-385-P	TCACGCGCATCAGGACAAGA	
OXA-48-like-131-F	TTGTGCTCTGGAATGAGAATAAGCAG	210 bp
OXA-48-like-340-R	CGGTGATTAGATTATGATCGCA	
OXA-48-186-P	GAACCAAGCATTTTTACCCGCA	
SPM-50-F	CGGATCATGTCGACTTGCCCT	200 bp
SPM-250-R	ACTACTTTCTTCGGCTTCATAGTC	
SPM-107-P	TCGTCACAGACCGCGATTTCT	

a,b *Primers for qPCR*.

### DNA preparation

To prepare DNA templates for the REBA-EAC assay, one colony from each frozen stock and one colony from each clinical isolate was suspended in 100 μL of DNA extraction solution (Optipharm, Osong, Republic of Korea). The bacterial suspension was boiled for 10 min. After centrifugation at 13,000 × g for 10 min, the supernatant was used as a source of DNA template.

### REBA-EAC primers and probes

**Figure 1:**
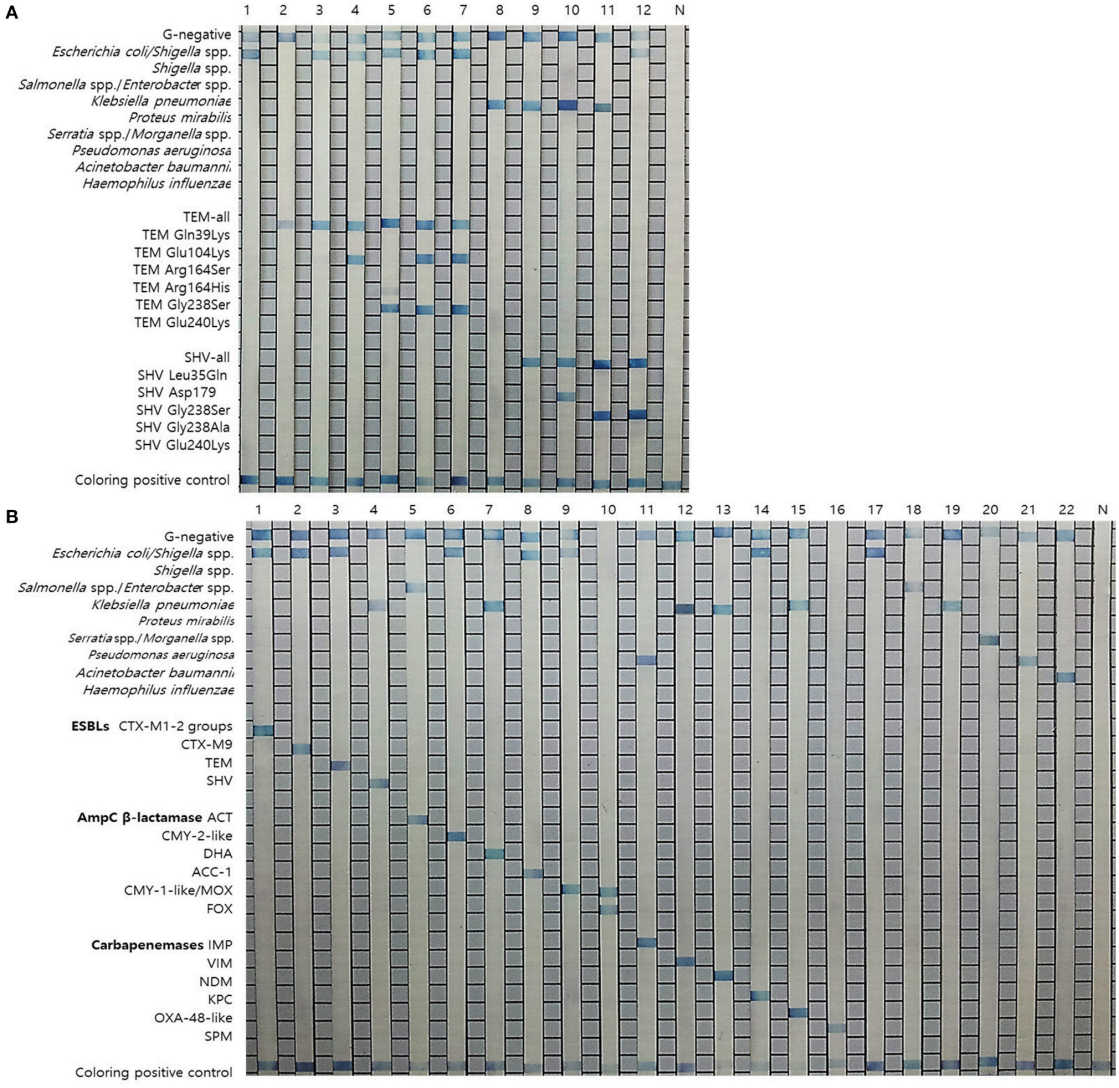
**Results of the REBA-EAC assay. (A)** Results of the REBA-EAC assay for the detection of TEM/SHV-ESBLs: Lane 1, *E. coli*; 2, TEM-1; 3, TEM-73; 4, TEM-ESBL (Glu104Lys); 5, TEM-ESBL (Gly238Ser); 6-7, TEM-52 and TEM-66 (Glu104Lys and Gly238Ser mixed); 8, *K. pneumoniae*; 9, SHV-1; 10, SHV-ESBL (Asp179Ala); 11-12, SHV-12 and SHV-2a (Gly238Ser). **(B)** Specificity of the REBA-EAC assay as tested with DNA from 16 EAC-producing and 6 reference bacterial species: Lane 1, CTX-M-1-2; 2, CTX-M-9; 3, TEM; 4, SHV; 5, ACT; 6, CMY-2-like; 7, DHA; 8, ACC-1; 9, CMY-1-like/MOX; 10, FOX; 11, IMP; 12, VIM; 13, NDM; 14, KPC; 15, OXA-48-like; 16, SPM; 17, *E. coli*; 18, *Enterobacter aerogenes*; 19, *K. pneumoniae*; 20, *Serratia marcescens*; 21, *P. aeruginosa*; 22, *A. baumannii*; N, negative control.

The 26 specific primers and probes were used for the detection of GNB (10 probes) and their antibiotic resistance genes (16 EAC gene probes) and an example of the result is shown in Figure 1. The sequences of the primers and probes were compared by nucleotide-nucleotide NCBI BLAST (BLASTn) searches to determine their sequence homologies. Primers and probes were selected by multiple alignments using the MultAlign program (http://multalin.toulouse.inra.fr/multalin/). All publicly available nucleotide sequences of the TEM and SHV alleles related to the ESBL-production phenotype (http://www.lahey.org/studies/) were retrieved from GenBank. Previous reports have concluded that 95% of all TEM-variants with an established ESBL phenotype have one or more amino acid substitutions at positions 39, 104, 164, 238, and/or 240; moreover, 77% of all ESBL SHV-variants have substitutions at positions 179, 238, and/or 240 (Cohen Stuart et al., 2010; Rubtsova et al., 2010). Based on these findings, 7 oligonucleotide probes (Figure 1) were designed to identify TEM-variants (one TEM-all probe and 6 probes for ESBL variants). Specifically, the probes were designed to detect the following ESBL-associated amino acid substitutions: Gln39Lys, Glu104Lys, Arg164Ser/His, Gly238Ser, and/or Glu240Lys. To identify SHV-variants, 6 probes (Figure 1) were designed (one SHV-all probe and 5 probes for ESBL variants). Specifically, the probes were designed to detect the following ESBL-associated substitutions: Leu35Gln, Asp179, Gly238Ser, and/or Glu240Lys (Cohen Stuart et al., 2010; Rubtsova et al., 2010).

### PCR

PCR was performed in a final reaction volume of 20 μL. Each reaction contained 2 × master mix (10 μL) (GeNet Bio, Daejeon, Republic of Korea), 5 μL primer mixture, and 5 μL sample DNA. The first 10 PCR cycles were comprised of denaturation at 95°C for 30 s, followed by annealing and extension at 60°C for 30 s. These 10 cycles were followed by 40 cycles of denaturation at 95°C for 30 s and annealing/extension at 54°C for 30 s. After the final cycle, samples were maintained at 72°C for 10 min to complete the synthesis of all strands. The amplicon was visualized as a single 230 bp band using a ChemiDoc system (Bio-Rad, Hercules, CA, USA).

### REBA-EAC assay

To validate the efficiency of the selected probes, target DNA samples amplified from EAC-producing strains were applied to REBA membrane strips spotted with the selected probes. Two genes (FOX and SPM) were synthesized (Bioneer, Daejeon, Republic of Korea) and amplified with custom PCR primers (FOX, F-5′-ATGCAACAACGACGTGCGTTCGC-3′ and R-5′-TGGCAAGCTCGGCCATGGTCAC-3′; SPM, F-5′-CGTTTTGTTTGTTGCTCGTTGCG-3′ and R-5′-TCAGCTGCTTTTATCCGGTCTTTC-3′), resulting in amplicons of 395 and 400 bp, respectively. The resultant products were mutagenized after subcloning into the pBHA vector. Two plasmids were extracted from the transformants, and the mutated sequences were confirmed by sequence analysis (CosmoGenetech, Seoul, South Korea).

For the REBA-EAC assay, hybridization and washing were performed according to the manufacturer's instructions. Each sample was tested in duplicate and all REBA experiments were performed twice. In brief, biotinylated PCR products were denatured at 25°C for 5 min in denaturation solution. The resulting single-stranded PCR products were transferred to hybridization solution and then incubated with REBA-EAC membrane strips at 55°C with shaking at 90 rpm for 30 min in the provided blotting tray. The strips were then washed twice with gentle shaking in 1 mL of washing solution for 10 min at 55°C, incubated at 25°C with a 1:2,000 dilution of streptavidin-alkaline phosphatase (AP) conjugate (Roche Diagnostics, Mannheim, Germany) in conjugate diluent solution (CDS) for 30 min, and finally, washed twice with 1 mL CDS at room temperature for 1 min. The colorimetric hybridization signals were visualized by the addition of a 1:50 dilution of the NBT/BCIP AP-mediated staining solution (Roche Diagnostics), and incubated until a color change was detected. Finally, the banding pattern was read and interpreted.

### Real time PCR (qPCR) assay

All phenotypic ESBL producers were screened by qPCR (CFX-96 qPCR system; Bio-Rad) to identify their non-ESBL and ESBL-carrying genes using primers specific for detecting predominant subtypes of CTX-M, TEM, and SHV. Next, the AmpC- and carbapenemase-carrying genes were analyzed using qPCR (CFX-96 qPCR system; Bio-Rad). Specifically, the presence of genes encoding ACT, CMY-2-like, DHA, ACC-1, CMY-1-like/MOX, FOX, IMP, VIM, NDM, KPC, OXA-48-like, and SPM was analyzed (Table 2). qPCR amplification was performed in a total volume of 20 μL, including 10 μL of 2 × Thunderbird SYBR master mix (Toyobo, Osaka, Japan), 2 μL primer mixture, 5 μL template DNA, and 3 μL sterile distilled water. Each qPCR assay included internal control DNA that was used to simulate pure nucleic acid extraction and high sample quality, thus monitoring the effect of PCR inhibitors in the reaction. Positive and negative controls were also included throughout the procedure. Template controls containing sterile distilled water were also incorporated in each assay. The thermocycling conditions were as follows: 95°C for 3 min, followed by 40 cycles of 95°C for 3 s and 60°C for 30 s. Each sample was tested in duplicate and all PCR assays were performed twice. The relative bacterial load was quantified by determining the cycle threshold (C_T_), i.e., the number of PCR cycles required for the fluorescence to exceed a value significantly higher than the background fluorescence. A positive result was interpreted as when the C_T_ value was less than 30 after fluorescence signal above background was observed.

### Sequence analysis

The PCR amplicons of all clinical isolates in this study were sequenced using an ABI 3730 automated DNA sequencer (Applied Biosystems, Foster City, CA, USA) and an ABI Prism BigDye Terminator (Applied Biosystems) system. The obtained sequences were compared with sequences in the NCBI GenBank database for species assignment.

### Blood culture samples

To evaluate the diagnostic performance of the REBA-EAC assay on blood culture samples, 200 positive blood culture (PBC), and 200 negative blood culture (NBC) samples were collected at WSCH from April 2015 to June 2015. Blood samples were drawn from the patient at the bedside. Three or two pairs of culture bottles were inoculated with the blood samples and then incubated in a BacT/Alert 3D (bioMérieux, Durham, NC, USA) or BACTEC FX (Becton Dickinson Diagnostic Systems, Spark, MD, USA) blood culture system for 5 days. If no bacterial growth was detected within 5 days, the blood culture was considered to be negative. When bacterial growth was noted, the culture sample was inoculated onto blood and MacConkey agar plates (Becton Dickinson) and cultured overnight at 37°C in a 5% CO_2_ incubator.

For the preparation of DNA templates from the PBC and NBC samples, 0.5 mL of blood suspension was withdrawn directly from the culture bottle. Next, 1 mL of phosphate-buffered saline (pH 8.0) was added and the sample was spun by centrifugation at 13,000 × g for 1 min. The supernatant was then removed, after which the pellet was resuspended in 1 mL of erythrocyte lysis buffer (Sigma, St. Louis, MO, USA) and spun by centrifugation at 13,000 × g for 1 min. This wash step was repeated twice. The pellet was then resuspended in DNA extraction solution, as described above for the clinical isolates. To confirm the positive results from the REBA-EAC assay, qPCR and sequence analyses were performed using the same DNA samples.

### Statistical analysis

All statistical analyses were performed using Prism 5 software (GraphPad, La Jolla, CA, USA) and SPSS statistics software version 21.0 (IBM, Armonk, NY, USA). The sensitivity, specificity, negative predictive value, and positive predictive value of the qPCR and REBA-EAC assays were analyzed by calculating the kappa coefficient of the predictive ability, its corresponding *P* value, and the 95% confidence interval (CI).

## Results

### Analytical sensitivity and specificity of the REBA-EAC assay

Twenty-nine EAC-producing strains were identified using the designated target probes in the REBA-EAC assay, whereas 128 non-EAC-producing clinical isolates yielded negative results by the REBA-EAC assay (data not shown). No cross-genus or cross-species reactivity was observed.

**Figure 2:**
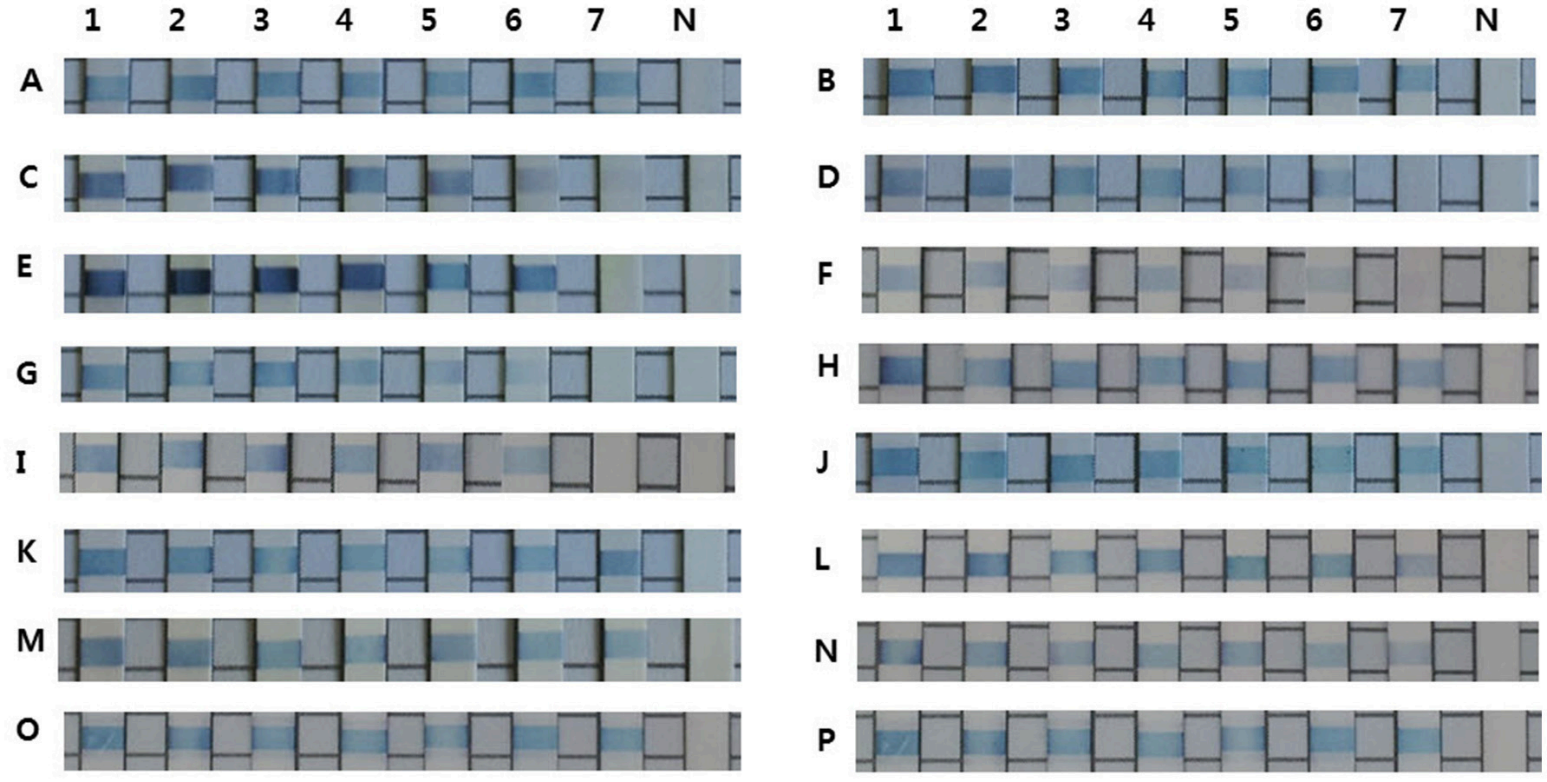
**Detection limits of the REBA-EAC assay for the 16 EAC gene probes**. Limits were determined using 10-fold serial dilutions of DNA samples from EAC-producing strains. A, CTX-M-1; B, CTX-M-9; C, TEM; D, SHV; E, ACT; F, CMY-2-like; G, DHA; H, ACC-1; I, CMY-1-like/MOX; J, FOX; K, IMP; L, VIM; M, NDM; N, KPC; O, OXA-48-like; and P, SPM. Lane 1, 10 ng; Lane 2, 1 ng; Lane 3, 100 pg; Lane 4, 10 pg; Lane 5, 1 pg; Lane 6, 100 fg; Lane 7, 10 fg, Lane 8, negative control.

The REBA-EAC assay was able to detect single nucleotide polymorphisms found in the most prevalent TEM- and SHV-type ESBLs, including Gln39Lys, Glu104Lys, Arg164Ser, Gly238Ser, and/or Glu240Lys in the TEMs, and Asp179Ala/Asn/Gly, Gly238Ser/Ala, and/or Glu240Lys in the SHVs. TEM-52, TEM-66 (Glu104Lys and Gly238Ser mixed), and SHV-12 (Gly238Ser), which were identified as TEM/SHV-ESBLs by the REBA-EAC assay, were also confirmed ESBL-associated amino acid substitutions by sequence analyses. The target DNA samples hybridized strongly with the probes derived from their targets and showed no cross-reactivity (Figure 1). The analytical sensitivities of the 16 target probes (4 ESBL, 6 AmpC, and 6 carbapenemase probes) were determined using a standard curve of 10-fold dilutions (10 ng, 1 ng, 100 pg, 10 pg, 1 pg, 100 fg, and 10 fg) of DNA extracted from EAC-producing strains. The detection limit of the REBA-EAC assay for each probe ranged from 100 fg to 10 fg DNA per reaction (Figure 2).

### Diagnostic performance of the REBA-EAC assay for clinical isolates

Based on the genus-specific or species-specific REBA-EAC assay probes, the overall concordance rate of the assay for species identification of the 327 Enterobacteriaceae isolates between the conventional identification test and the REBA-EAC assay was 100%. Of the 327 ESBL-phenotypically confirmed isolates, 308 isolates (94.2%) were positive by the REBA-EAC assay for ESBL genes, 16 *K. pneumoniae* isolates had only AmpC plasmid-mediated DHA genes, and three *E. coli* isolates did not have any EAC genes by the REBA-EAC assay (Table 3). Among the 324 isolates with at least one EAC gene, exclusive ESBL-producing isolates accounted for up to 70.0% of all EAC-producers (one-type ESBL producer, 154 isolates; two-type ESBL co-producer, 73 isolates), followed by 23.1% for ESBL and AmpC β-lactamase; 4.9% for AmpC β-lactamase; 1.5% for ESBL and carbapenemase; and 0.3% for ESBL, AmpC, and carbapenemase (Table 3). Overall, 80.2% (247/308) of ESBL-producing isolates were the CTX-M type, with CTX-M-9 group (57.5%) being the most prevalent ESBL gene. For the TEM and SHV genes, the REBA-EAC assay was able to differentiate between non-ESBL TEM/SHV, and their ESBL derivatives for all isolates. Among the 144 pan-TEM-positive isolates tested by qPCR, 104 (72.2%) were positive for G104L (TEM-3, -22, -52, -66), G238S, and/or G240L (TEM-22, -46, -52). Moreover, remaining 40 (27.8%) isolates, which were negative for G104L, G238S, and/or G240L, were considered to be non-ESBL producers by both the REBA-EAC assay and sequence analysis. The SHV-ESBL specific amino acid substitution, G238S, was detected in 31 (26.1%) of the 119 SHV-producing *Klebsiella* spp., and was confirmed in the SHV-2a, -5, and -12 genes by sequence analysis (Table 4).

**Table 3:** **REBA-EAC assay results for the detection of ESBLs, AmpC β-lactamases, and carbapenemases in the 327 ESBL-phenotypically confirmed isolates**.

**ESBL-phenotypically confirmed isolate (no. of isolates)**	**REBA-EAC assay**	
	**GN-ID (no. of isolates)**	**β-lactamase content (no. of isolates)**
*Escherichia coli* (196)	*E. coli* (196)	CTX-M only (109)
		CTX-M + TEM-type ESBL (68)
		TEM-type ESBL (5)
		CTX-M + CMY2 (4)
		CTX-M + DHA (2)
		CTX-M + NDM (2)
		CTX-M + ACT (1)
		CTX-M + KPC (1)
		CTX-M + VIM (1)
		ND (3)
*Klebsiella pneumoniae* (120)	*K. pneumoniae* (120)	CTX-M + DHA (58)
		TEM-type ESBL (11)
		CTX-M only (10)
		SHV-type ESBL (9)
		CTX-M + CMY2 (7)
		CTX-M + SHV-type ESBL (5)
		CTX-M + DHA + CMY2 (2)
		CTX-M + IMP (1)
		CTX-M + DHA + NDM (1)
		DHA only (16)
*Proteus mirabilis* (4)	Gram negative (4)	CTX-M (4)
*Klebsiella oxytoca* (3)	*K. oxytoca* (2)	SHV-type ESBL (2)
	*K. oxytoca* (1)	CTX-M (1)
*Enterobacter* spp. (3)	*Enterobacter* spp. (2)	CTX-M (2)
	*Enterobacter* spp. (1)	CTX-M + ACT (1)
*Providencia stuartii* (1)	Gram negative (1)	CTX-M (1)

*ND, not detected*.

**Table 4:** **Comparison of the performances of the REBA-EAC assay versus sequence analysis for detecting β-lactamase genes in the 327 ESBL-phenotypically confirmed isolates**.

**Class of β-lactamase (no. of isolates)**	**Type of** β**-lactamase gene**		**Microorganism**	**No. of isolates**
	**REBA-EAC assay**	**Sequence analysis**		
ESBL (382)	CTX-M-1/2	CTX-M-1	*E. coli*	97
	CTX-M-1/2	CTX-M-1	*K. pneumoniae*	3
	CTX-M-1/2	CTX-M-1	*K. oxytoca*	1
	CTX-M-1/2	CTX-M-1	*E. aerogenes*	1
	CTX-M-9	CTX-M-9	*E. coli*	86
	CTX-M-9	CTX-M-9	*K. pneumoniae*	49
	CTX-M-9	CTX-M-9	*P. mirabilis*	4
	CTX-M-9	CTX-M-9	*E. cloacae*	2
	CTX-M-9	CTX-M-9	*P. mirabilis*	4
	CTX-M-1/2 + CTX-M-9	CTX-M-1 + CTX-M-9	*E. coli*	2
	CTX-M-1/2 + CTX-M-9	CTX-M-1 + CTX-M-9	*K. pneumoniae*	1
	TEM	TEM-3	*K. pneumoniae*	3
	TEM	TEM-22	*E. coli*	21
	TEM	TEM-22	*K. pneumoniae*	5
	TEM	TEM-46	*E. coli*	9
	TEM	TEM-46	*K. pneumoniae*	5
	TEM	TEM-52	*E. coli*	25
	TEM	TEM-52	*K. pneumoniae*	10
	TEM	TEM-66	*E. coli*	21
	TEM	TEM-66	*K. pneumoniae*	5
	SHV	SHV-2a	*K. pneumoniae*	14
	SHV	SHV-5	*K. pneumoniae*	5
	SHV	SHV-12	*K. pneumoniae*	10
	SHV	SHV-12	*K. oxytoca*	2
AmpC β-lactamase (92)	DHA	DHA-1	*K. pneumoniae*	61
	DHA	DHA-23	*K. pneumoniae*	8
	DHA	DHA-15	*K. pneumoniae*	5
	DHA	DHA-3	*K. pneumoniae*	1
	DHA	DHA-1	*E. coli*	2
	CMY-2-like	CMY-2	*K. pneumoniae*	7
	CMY-2-like	CMY-2	*E. coli*	4
	DHA+CMY-2-like	DHA+CMY-2	*K. pneumoniae*	2
	ACT	ACT-1	*E. coli*	1
	ACT	ACT-1	*E. aerogenes*	1
Carbapenemase (6)	NDM	NDM-1	*E. coli*	2
	NDM	NDM-1	*K. pneumoniae*	1
	KPC	KPC-18	*E. coli*	1
	VIM	VIM-2	*E. coli*	1
	IMP	IMP-1	*K. pneumoniae*	1

### Diagnostic performance of the REBA-EAC assay for blood culture samples

**Figure 3:**
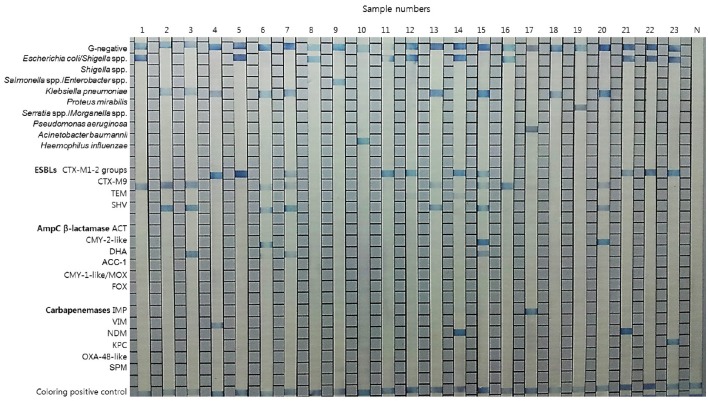
**Representative results for the REBA-EAC assay performed with clinical isolates and positive blood culture samples**. Lanes 1–7, 11–17, and 20–23, EAC-producing strains; lanes 8–10, 18, and 19, non-EAC-producing strains; N, negative control.

Typical results of the REBA-EAC assay using blood culture samples are shown in Figure 3. Among the 200 PBC samples, 120 (60%), 73 (36.5%), and 7 (3.5%) samples contained GPB, GNB, and *Candida* species by the conventional identification test, respectively. None of the GPB or *Candida* species were detected by the REBA-EAC assay (Table 5). Based on the genus-specific or species-specific REBA-EAC assay probes, the sensitivity, specificity, positive predictive value, and negative predictive value of the REBA-EAC assay for GNB identification in blood culture specimens were 100% (95% CI 0.938–1.000, *P* < 0.001), 100% (95% CI 0.986–1.000, *P* < 0.001), 100% (95% CI 0.938–1.000, *P* < 0.001), and 100% (95% CI 0.986–1.000, *P* < 0.001), respectively. Among the 57 Enterobacteriaceae isolates, only 12 (12/57; 21.1%) isolates were phenotypically confirmed as ESBL producers, whereas 16 (16/57; 28.1%) isolates were confirmed as carrying EAC genes by both the REBA-EAC assay and sequence analysis (Table 6). Eleven of the twelve (91.7%) phenotypically ESBL-positive isolates also showed positive results by the REBA-EAC assay, with CTX-M type genes (8 CTX-M-1 and 3 CTX-M-9) being the most prevalent ESBL genes. One *Pseudomonas aeruginosa* and five Enterobacteriaceae isolates (3 *Enterobacter cloacae*, 1 *Citrobacter freundii*, and 1 *Providencia stuartii*) were positive for an IMP and AmpC genes (ACT, CMY, or DHA), respectively, by the REBA-EAC assay. This finding was confirmed by qPCR and Sanger sequencing.

**Table 5:** **Comparison of species identification results obtained using the conventional method versus the REBA-EAC assay on 400 blood culture samples**.

**Conventional method (no.)**	**REBA-EAC assay no. (%)**	
	**Positive**	**Negative**
Positive blood culture (200)	73 (36.5)	127 (63.5)
Gram positive (120)	0 (0)	120 (100)
Gram negative (73)	73 (100)	0 (0)
*Candida* spp. (7)	0 (0)	7 (100)
Negative blood culture (200)	0 (0)	200 (100)
Total (400)	73 (18.3)	327 (81.7)

**Table 6:** **Comparison of the results of phenotypic versus molecular assays for the detection of β-lactamases in 73 Gram-negative bacteria isolates from blood culture samples**.

**Microorganism**	**No. (%) of isolates**	**Detection of** β**-lactamases, no. (%) of isolates**							
		**Phenotypic assay**		**REBA-EAC assay**		**qPCR**		**Sequence analysis**	
		**Positive**	**Negative**	**Positive**	**Negative**	**Positive**	**Negative**	**Positive**	**Negative**
*E. coli*	33 (45.2)	11 (33.3)	22 (66.7)	10^a^ (30.3)	23 (69.7)	20^b^ (60.6)	13 (39.4)	10^a^ (30.3)	23 (69.7)
*Klebsiella* spp.	14 (19.1)	1^c^ (7.1)	13 (92.9)	1^c^ (7.1)	13 (92.9)	12^b^ (85.7)	2 (14.3)	1^c^ (7.1)	13 (92.9)
*Enterobacter* spp.	5 (6.8)	0 (0)	5 (100)	3^d^ (60)	2 (40)	3^d^ (60)	2 (40)	3^d^ (60)	2 (40)
*Providencia* spp.	2 (2.7)	0 (0)	2 (100)	1^e^ (50)	1 (50)	1^e^ (50)	1 (50)	1^e^ (50)	1 (50)
*Citrobacter freundii*	1 (1.4)	0 (0)	1 (100)	1^f^ (100)	0 (0)	1^f^ (100)	0 (0)	1^f^ (100)	0 (0)
*Serratia marcescens*	1 (1.4)	0 (0)	1 (100)	0 (0)	1 (100)	0 (0)	1 (100)	0 (0)	1 (100)
*Proteus mirabilis*	1 (1.4)	0 (0)	1 (100)	0 (0)	1 (100)	0 (0)	1 (100)	0 (0)	1 (100)
*Campylobacter* spp.	1 (1.4)	NA	NA	0 (0)	1 (100)	0 (0)	1 (100)	0 (0)	1 (100)
*Acinetobacter* spp.	7 (9.6)	NA	NA	0 (0)	7 (100)	0 (0)	7 (100)	0 (0)	7 (100)
*Pseudomonas aeruginosa*	6 (8.2)	NA	NA	1^g^ (16.7)	5 (83.3)	1^g^ (16.7)	5 (83.3)	1^g^ (16.7)	5 (83.3)
*Sphingomonas paucimobilis*	1 (1.4)	NA	NA	0 (0)	1 (100)	0 (0)	1 (100)	0 (0)	1 (100)
*Burkholderia cepacia*	1 (1.4)	NA	NA	0 (0)	1 (100)	0 (0)	1 (100)	0 (0)	1 (100)

a *Isolates with CTX-M gene (8 CTX-M-1 and 2 CTX-M-9)*.

b *Some of isolates with non-ESBL type TEM or SHV genes were included in the positive results*.

c *Isolate with CTX-M gene (CTX-M-9)*.

d *Isolates with ACT gene*.

e *Isolate with DHA gene*.

f Isolate with CMY gene

g *Isolate with IMP gene*.

*NA, not available*.

## Discussion

Presently, antimicrobials are usually selected empirically for the initial treatment of bacteremia when a bloodstream infection is strongly suspected (Park et al., 2014). Furthermore, since the antimicrobial susceptibility results usually cannot be provided in < 48–72 h, clinicians continue to rely on Gram stain results for selection of an appropriate antibiotic treatment for patients with PBCs (Uehara et al., 2009). Rapid and accurate EAC detection is crucial for effective infection control measures and for the selection of appropriate antimicrobial therapy, because the emergence of EAC-producing strains is a vital factor in the treatment of infections associated with sepsis. Like other mechanisms of antibiotic resistance, the genes for EACs are most often encoded on plasmids that can be readily transferred between bacteria (Haenni et al., 2014). The use of semiautomated systems for the identification and antimicrobial susceptibility testing of GNB is now routine practice in many laboratories. Compared to more recently developed molecular tests, traditional phenotypic susceptibility results usually take an additional 1–2 days after a PBC is reported. Since phenotypic detection of ESBLs, AmpCs, and carbapenemases is time-consuming and the results may be difficult to interpret, a faster and more accurate detection method is highly desirable for routine use in clinical laboratories (Cohen Stuart et al., 2010). In general, commercially available molecular based tests have been developed for one of ESBL (CTX-M types, TEM, and SHV), AmpC β-lactamase (CMY-2-like, DHA, FOX, ACC-1, ACT/MIR, and CMY-1-like/MOX), and carbapenemase (VIM, IMP, NDM, KPC, and OXA-48-type) genes (Diekema and Pfaller, 2013). In comparison, REBA-EAC assay can provide information about GNB identification and broad range of clinically relevant antibiotic resistance genes in GNB.

Although the prevalence of carbapenemase-producing Enterobacteriaceae strains is still very low (< 1%) in Korea, various types of carbapenemases have been identified (Bae et al., 2015). In this study, ESBL and AmpC β-lactamase co-production was seen in 23.1%; ESBL and carbapenemase co-production in 1.5%; and ESBL, AmpC β-lactamase, and carbapenemase co-production in 0.3% of the phenotypically confirmed ESBL-producing Enterobacteriaceae isolates. Several studies have demonstrated that AmpC β-lactamase with ESBLs causes treatment failure of cefepime and that inadequate antimicrobial therapy is an independent risk factor for mortality or microbiological failure in severely ill patients with life-threatening infections (Fraser et al., 2006; Park et al., 2010). Therefore, rapid and accurate identification of drug resistance genes directly from PBCs is critical for the selection of appropriate antibiotics in the treatment of bacterial infections.

In this study, we confirmed that the REBA-EAC assay is a highly sensitive and specific probe-based method that provides rapid results and can detect multiple species and drug-resistant genes simultaneously (Ajbani et al., 2011). The REBA-EAC assay also showed complete agreement with the conventional identification test and sequence analysis for the detection of ESBL (CTX-M groups, TEM-ESBL derivatives, SHV-ESBL derivatives) and non-ESBL (TEM and SHV) genes. For qPCR, TEM/SHV show positive results for both ESBL and non-ESBL, which makes it difficult to differentiate between them. However, as REBA-EAC assay detects one or more amino acid substitutions in targeted region, it can discriminate between ESBL TEM/SHV derivatives and non-ESBL TEM/SHV types. In general, our results are similar to previous assessments of REBA assay (sensitivity of 95–99% and specificity of 100%) (Kim et al., 2016; Wang et al., 2016) and other methods such as microarray-based check-points assay (sensitivity of 94–95% and specificity of 83–100%) (Cohen Stuart et al., 2010; Card et al., 2013) and MALDI-TOF MS-based assay (sensitivity of 97–99% and specificity of 94–99%) (Bork et al., 2015; Chong et al., 2015). Moreover, the performance of the REBA-EAC assay was better than the *E*-test (sensitivity of 71% and specificity of 73%) (Garrec et al., 2011). We also found that CTX-M type genes (CTX-M-1 and CTX-M-9) were the most prevalent ESBL-encoding genes. These genes were detected in almost all of the ESBL-producing Enterobacteriaceae (in particular, 48.4% of all *E. coli* isolates), whereas ESBL-type TEM and SHV genes were present in 20.4 and 6.1% of the isolates, respectively. These findings are in agreement with other recent studies showing that CTX-M type genes are the ESBL genes most frequently expressed by Enterobactericeae (Cantón et al., 2012). In many Enterobacteriaceae, AmpC expression is low but inducible in response to β-lactam exposure (Haenni et al., 2014). Using the REBA-EAC assay, we observed various types of AmpC β-lactamases (DHA, CMY-2, DHA, and ACT) in different Enterobacteriaceae species: CMY-2 in *E. coli*, DHA-1 in *K. pneumoniae* or *E. coli*, and ACT in *Enterobacter aerogenes*. In particular, DHA-producing *K. pneumoniae* isolates were predominant in Enterobacteriaceae, a finding that is similar to the results reported in other studies (Park et al., 2010).

Our study confirmed that REBA-EAC assay can be applied directly in blood culture samples. As shown in Table 5, the results of conventional identification test and REBA-EAC assay were completely concordant in species identification. Furthermore, REBA-EAC assay detected isolates harboring AmpC or carbapenema genes (1 *Pseudomonas aeruginosa*, 3 *Enterobacter cloacae*, 1 *Citrobacter freundii*, and 1 *Providencia stuartii*).

One potential limitation of our study compared to previous studies is the limited number of samples, since the study was carried out at a single institution. Neither the AmpC β-lactamase ACC-1, CMY-1-like/MOX, or FOX genes, nor the carbapenemase OXA-48-like gene, were detected in clinical isolates of this study. Moreover, since AmpC and carbapenemase genes are less prevalent than ESBL genes in Korea, we confirmed and included only ESBL-phenotype isolates in screening. Thus, there is a possibility that some of the non-ESBL isolates producing AmpC and/or carbapenemase could not be identified. Therefore, larger studies are required to confirm our results and limitations.

In conclusion, the recently developed REBA-EAC assay is an accurate, rapid, and convenient tool for identifying GNB. This assay can also discriminate ESBL, AmpC β-lactamase, and carbapenemase genes and directly detect pathogen species from PBC samples in only 2 h. Therefore, the REBA-EAC assay can provide essential information that can accelerate therapeutic decisions for earlier appropriate antibiotic treatment in the acute phase of pathogen infection.

## Author contributions

YU, WS, JBK, and HL designed the experimental strategy. HW and GY performed the experiments. HW, GY, and JK analyzed and interpreted the data. HW and GY wrote the paper with input from all co-authors. All authors have read and approved the final version of the manuscript.

### Conflict of interest statement

The authors declare that the research was conducted in the absence of any commercial or financial relationships that could be construed as a potential conflict of interest.
